# 

**Published:** 1828-06

**Authors:** 


### MONTHLY LIST OF MEDICAL BOOKS.

[*Medical Works cannot be entered on this List except a copy be sent for the purpose; the titles of Books having frequently been transmitted to us, as published, which have not appeared for weeks, or even months, after.*]

A Treatise on Gout, Apoplexy, Paralysis, and Disorders of the Nervous System. By A. RENNIE, Surgeon, &c.—8vo. pp. 213. Burgess and Hill, London, 1828.

Hints to Young Medical Officers of the Army on the Examination of Recruits, and respecting the feigned Disabilities of Soldiers; with Official Documents, and the Regulations for the Inspection of Conscripts for the French and Prussian Armies. By HENRY MARSHALL, Surgeon to the Forces.—8vo. pp. 224. Burgess and Hill, London, 1828.

Observations on Strictures of the Rectum, and other Affections of the Intestinal Canal; including Hæmorrhoidal Tumors (called Piles), accompanied with the Mode of Treatment by the late W. WHITE, Esq. By SAMUEL WHITE, Member of the Royal College of Surgeons, London. Fifth Edition. —8vo. pp. 110, and Plates. Hunt, Bath, 1828.

**A Clinical Lecture delivered to the Students of Surgery in the Royal Infirmary of Edinburgh, at the conclusion of the Winter Course for 1827-28. By George Ballingall, M.D. F.R.S.E. &c.—4to. pp. 29. Edinburgh, 1828.**

**An Investigation of the Properties of the Thames Water. By William Lambe, M.D. Fellow of the Royal College of Physicians.—8vo. pp. 65. T. Butcher, and T. and G. Underwood, London, 1828.**

**A Dissertation on the Nature and Properties of the Malvern Water, and an Enquiry into the Causes and Treatment of Scrofulous Diseases and Consumption; together with some Remarks upon the Influence of the Terrestrial Radiation of Caloric upon local Salubrity. By William Addison, Surgeon. —8vo. pp. 192. Callow and Wilson, London, 1828.**

### METEOROLOGICAL JOURNAL,

### NOTICES.

***We regret that we cannot insert the Paper signed "Caution;" for, though we agree in the prudence of aiding the invitation from the Russian government to our medical men to serve in their army, we dissent from the reflections against the medical department at home.***

### INDEX

TO THE

FOURTH VOLUME OF THE NEW SERIES

OF THE

### London Medical and Physical Journal.

PAGE

ABERDEEN New Dispensary, cases treated at the . . 26
Acid in Iceland Moss . . 369
Alkali in Hemlock . . 83
Amputation at the Hip-joint . 80
Anatomy, Manual of, by H. M. Edwards, translated by Coulson, (analysis of) . . 54
Anatomy, Sir James Mackintosh's speech on the present state of 554
Anchylosis, Dr. Barton's operation for . . . 77
———, case of, cured by operation . . . 457
Aneurism, diffuse . . 25
———, Mr. Wardrop's proposed method of tying the vessel beyond the tumor . 172
———, Inguinal . . 328
——— of the Heart, in the case of Talma . . 163
———, Femoral and Popliteal, external Iliac tied . . 325
Animals, methods of procuring either sex . . . 161
Anterior Tibial Artery, wound of 547
Anus, artificial, case of . 164
Axillary Aneurism of the right side, Subclavian tied . 135

Balsam of Copaiba, mode of correcting the taste of . . 553
Bees, death produced by their sting . . . . 164
Belladonna, a preventive of Scarlatina . . . 67
Beriberi, nature and treatment of . . . . 197
Bill, to regulate the care of insane persons . . . 557
Bills of Mortality . . 182

PAGE

Bite of the Viper, observations on the . . . 73
Black Vomit . . . 100
Blood, spots of . . 366
———, iron contained in the . 82
Bones, pathology of the 403, 4[illegible]0
Bronchia, Diseases of, analysis of M. Laennec on . . 145
Bronchotomy, causes of the fatal terminations in cases of . 249
British Opium, remarks on . 505
Buboes, on the application of Tobacco Ointment to . 506
Burns, cases of, treated with Cotton . . . 511

Carcinoma of the Stomach, case of . . . . 230
——— of the Cæcum . 227
Cataract, hereditary disposition to . . . . 354
———, varieties of . . 89
Chorea cured by Iodine . 454
Circulation, observations on the 1
Clinical Report of Chest Affection 285
Cold water in Salivation, use of 76
College of Physicians, their By-Laws . . . 371
Comparative Anatomy, M. Carus's, translated by Gore, (analysis of) . . . 40
Concussion followed by Extravasation . . . 219
Conium, new alkali in . . 83
Constipation, observations on . 4[illegible]4
Copaiba, volatile oil of . 2[illegible]5
Cow-Pox, Mr. Edmonston on (analysis of) . . . 526
Cynanche Parotidœa, use of Iodine in . . . 73

PAGE
Death from Lead, cases of . 453
——— from the sting of Bees . 164
Delirium Tremens, Dr. Coates on 356
Dentition, remarks on . . 353
Diabetes Mammané, case of . 538
Disease of the Elbow-joint, and removal of part of the Bones 313
Dislocations of the Vertebra, Mr. Lawrence on . . 74
Distinctions between Syphilis and Phagedena . . 77
Drowning, signs of . . 170

Ecchymosis, spontaneous . 164
Elbow-joint, disease of, and removal of part of the Bones . 313
Empirics, proceedings against, in the reign of Edward VIth. 84
Encephalitis cured by Acupuncturation . . 538
Epilepsy, case of, cured by a severe Burn . . . 355
———, Artimesia Vulgaris in ib.
Ergot of Rye, notice respecting the . . . . . 367
——— its use in Hydatids of the Uterus . . . 545
Erysipelas and Injury of the Head, successfully treated by bleeding . . . 223
———, treated with leeches and stimulants . . 226
Essay on Diseases of the Jaws, Mr. Koecker on . . 582
Evolution, spontaneous, of the Fœtus . . . 102
Exfoliation of a portion of the Temporal Bone, from a blow 217
Extirpation of Ovarian Tumors, remarks on the . . 175
Extra-uterine Fœtation, case of 378

Fever, observations on . 166, 385
———, cases of, treated at the Glasgow Infirmary . . 417
———, on the propagation of . 68
Foramen Ovale, open, case of 353
Fracture of the Skull, with Malformation of the Brain . 221
——— with Depression, successfully treated without operation . . . . ib.
——— of the Skull, with depression, case of . . 516
——— into the Knee-joint, successfully treated . . 517

Gastrocnemii Muscles, congenital absence of . . 161
PAGE
General View of the present State of Lunatics, by Sir A. Halliday, (analysis of) . 427
Glossitis, cases of . . 28
Gun-shot Wound, case of, in which five-sixths of the seat of injury was destroyed, the limb being still preserved . 508

Hair, remarkable quantity of, in a man . . . 248
Hernia Cerebri, case of . 218
——— in an infant, requiring operation . . . 421
———, Scrotal, remarkable case of . . . 423
———, Strangulated, cases of 125
———, case of, in which the gut was ruptured by a blow on the sac . . . 515
———, Strangulated, successfully treated with a Tobacco Enema . . . 516
Hip-joint, amputation at the . 80
Hydatids of the Uterus, successfully treated with Ergot . 545
Hydrophobia, proposal to treat by means of heated air . 497

Imputation of Poisoning—detection of conspiracy . . 361
Indelible writing ink . . 83
Indigestion, Dr. Uwins on (analysis of) . . . 58
Iliac, common, successful ligature of the . . . 463
Injury of the Spine . . 26
——— of the Spine, case of, in which some phenomena resembling electricity were observed . . . 546
Ink, indelible . . . 83
Insane persons, Bill to regulate the care of . . . 557
Intus-susception, fatal case of 504
Iodine, its use in enlargements of the Liver and Spleen . 234
———, its use in Cynanche Parotidœa . . . 73
——— in Gout . . 537
Ipecacuanha, use of, in Menorrhagia, Dr. Osborne on (analysis of) . . . 520
Iron contained in the Blood . 82
Irritability of the Sensitive Plant 468

Jaundice, Thoughts on the Pathology and Treatment of . 478

PAGE
Kidney Bean passed by the Urethra . . . . 550
Knee-joint, Fracture into, successfully treated . . 517

Lachrymal Duct, M. Dupuytren's method of treating Strictures of the . . . 254
Larynx, case in which the passage was restored after having become nearly obliterated . 248
Libel against the Editor of the London Medical and Physical Journal . . . 257
Life, observations on . . 9
Liver and Spleen, enlargements of, treated with Iodine . 234
London Pharmacopœia, Grammatical Introduction to the . 352
Lungs, Diseases of the, by M. Laennec, (analysis of) . 346

Malignant Tumor within the Thorax, case of . . 231
Means of promoting the growth of the Hair . . . 470
Medicus, his reply to Dr. Gregory . . . 109
Melancholia, with Loss of Voice, cured by the actual Cautery 537
Melanosis, case of . . 34
Meteorological Journals, 88, 184, 280, 376, 472, 560
Method of dissecting and preparing the Bodies of Animals . 451
Midwifery in different countries 370
———, cases in . . 501
Monthly Reports of Prevalent Diseases 84, 181, 371, 471, 553
——— lists of Books, 88, 184, 375, 471, 559
Mortality and Management of Children, Mr. Robertson on, (analysis of) . . . 142
Moxa, cases of Epilepsy and Paralysis in which it was successfully applied . . 473
Mucous Membrane of the Stomach and Intestines, inquiry concerning . 113, 208, 304

Nævi, treatment of, by ligature 178
Nævus, case of, cured by Vaccination . . . 425
Narcotine, method of preparing 256
Nasal Polypi cured by the saffronised Tincture of Opium 76
Nature and Properties of Living Animals, Disquisition concerning, (analysis of) . . 438

PAGE
Necrosis, Professor Smith on . 461
Nitrate of Silver, on the external application of . . 458
———, on the use of, by Mr. Ryall, analysis of . 518
Notices . . 88, 376, 560

Observations on Fever . 185, 291
Oleum Ricini, singular effect of 553
Opium, British, remarks on . 505
Oxalic Acid, case of poisoning with, successfully treated . 517

Paraplegia, Mr. Earle on . 65
———, case of . . 37
Paruria Erratica, case of . 538
Pathological Researches on the Brain and Spinal Cord, by Dr. Abercrombie, (analysis of) 237, 330
Peripneumonia of Children, Dr. Cuming on (analysis of) . 521
Petition of the Physicians to the Legislature . . . 84
Phagedenic Ulceration . 34
Physiology of the Iris . . 281
Plates of Lead, application of, to Wounds . . . 550
Poisoning with Arsenic, case of 363
———, false imputation of, detected . . . 361
——— with Oxalic Acid, case of, successfully treated . 517
Polypi of the Nose cured by the saffronised Tincture of Opium (Prussian Pharm.) . . 76
Practical Observations on Insanity and the Treatment of the Insane . . . 427
Pregnancy, Dr. Merriman on the duration of . . . 81
Premature growth, case of . 161
——— labour, proposed method of bringing on . . 176
Pulsating Tumors, cases of . 319

Quinæ Sulphas, on large doses of 166
Quina, Sulphate of, method of preparing it without Alcohol 255

Recovery from Drowning . 470
Reduction of a Shoulder which had been dislocated seven months . . . 551
——— of a Femur which had been dislocated for three months . . . ib.
——— of Humerus which had been dislocated ten months and a half . . . 552

PAGE
Reduction, convenient mode of effecting, in Dislocations of the Humerus . . 552
Remarks on the conduct of the Editor of the Lancet towards the Editor of this Journal . 278
Remarkably hairy man . 248
Reply to Medicus, on constitutional inaptitude to Small-Pox 14
Report, Medical, of Lady Jane Seymour's illness . . 84
——— of the National Vaccine Establishment . . 374
——— on the presumed Violation of a Child . . 449
Rheumatism, terminating in ulceration of the cartilages . 311
Rupture of the Bowel in a hernial sac . . . . 515

Salivation cured by cold water 76
Sensitive Plant, irritability of the 468
Seton, on the use of, in diseases of the Bones and Joints . 461
Skull, Fracture of, with depression, cases of . . 129
——————, with depression . . . . 516
Smell, peculiarities in the sense of . . . . . 62
Spina Bifida, cured by puncture 177
Spinal disease, case of . . 424
Spleen and Liver, enlargements of, treated with Iodine . 234
———, part of, excised . 549
Spontaneous evolution of the Fœtus . . . 102
Spots of Blood . . 366
Sprains, Memoir on . . 391
Stammering, case of, cured . 553

PAGE
Strangulated Hernia, cases of 125
Stricture of the Rectum, analysis of Mr. Salmon on . . 136
Sudden Death . . . 29
Suffocation, cases of . . 122

Talma's Heart . . . 163
Tic Douloureux, Sir H. Halford on . . . . 452
Tobacco Enema, successfully used in Strangulated Hernia 516
Tobacco, its efficacy in Cynanche Tonsillaris . . 546
Tracheotomy, case of . . 426
Transactions of the Fellows and Licentiates of the Dublin College of Physicians (analysis of) 518
Trial of Thomas Wakley for Libel . . . 257
Tumor, case of large, on the forehead . . . 301

Uterine Hemorrhage, case of . 107
Uterus, Laceration of, remarks on . . . . 367
Urethra, ruptured, case of . 315
Urine, Retention of . . 318

Vaccination in Turkey . 13
——— in Africa . . 194
——— and Small-Pox, identity of . . . 508
Vein, inflammation of a, case of 22
Verdict in the cause Rolfe *versus* Stanley . . . 357
Vicarious Urinary Discharge, case of . . . 542
Violation of a Child, report on the supposed . . 449

### ORIGINAL PAPERS AND CASES.

PAGE

### PHYSIOLOGY.

Observations on the Phenomena of the Circulation. By J. H. James, M.D. 1
On Life. By Sir George Smith Gibbes . . . . 9
On the Physiology of the Iris. By Egerton A. Jennings, Esq. . 281

### FEVER.

Observations on Fever. By Dr. Bow, of Alnwick . 185, 291, 385
Cases of Fever, treated at the *Infirmary of Philadelphia* . . 412
Case of Tertian Intermittent. By H. R. Oswald, Esq. . . 302

### PATHOLOGY OF THE BONES.

A Dissertation on the Pathology of the Bones, with illustrative Cases; among others, a Case of the Removal of Carious Ribs. By Wm. A. M'Dowell, M.D. . . . . . . 403, 490

### INJURIES OF THE HEAD.

Case of Extravasation of the Brain . . . . . . 34
Cases of Compound Fracture of the Skull, with Depression. *St. Bartholomew's Hospital.* With Observations by B. Travers, F.R.S. . 129
Exfoliation of a Portion of the Temporal Bone, from a Blow. *Middlesex Hospital* . . . . . . . . 217
Case of Hernia Cerebri. *Ditto* . . . . . 218
——— Concussion, followed by Extravasation. *St. Bartholomew's Hospital* . . . . . . . . 219
——— Fracture with Depression, successfully treated without Operation. *St. George's Hospital.* . . . . . . 221
——— Fracture of the Skull; Malformation of the Brain. *St. Thomas's Hospital* . . . . . . . . . ib.
Fracture of the Skull, with Depression, in a Child, successfully treated, at the *Middlesex Hospital* . . . . . . 516

### CATARACT.

On the Varieties of Cataract; their Causes, Formation, and Cure. By S. J. Stratford, Esq. Assistant Surgeon 72d Highlanders . 89

### SPINAL DISEASE.

Case of Injury of the Spine . . . . . . 26
——— extensive Spinal Disease . . . . . 424

### PARAPLEGIA.

Case of Paraplegia. By Charles Thomas, M.D. . . . 37

### DISEASES OF THE JOINTS.

Case of Rheumatic Inflammation, terminating in Ulceration of the Cartilages. *St. George's Hospital* . . . . . 311
Disease of the Elbow Joint, with Removal of Part of the Bones. *La Charité* . . . . . . . . . 313

### STOMACH AND INTESTINES.

Inquiries into the Healthy and Diseased Appearances of the Mucous Membrane of the Stomach and Intestines. By W. E. Horner, M.D. 113, 208, 304

PAGE

### ERYSIPELAS.

Case of Erysipelas, treated at the *Middlesex Hospital* . . . 17
——— Inflamed Vein and extensive Phlegmonous Erysipelas of the Arm, following Venesection, treated by Incisions. *St. Bartholomew's Hospital* . . . . . . . . . . 22
——— Erysipelas, with Sloughing of the Cellular Membrane, successfully treated by Incisions. *Ditto* . . . . . 224
——— Phlegmonous Erysipelas—Incisions—Fatal Hemorrhage. *Ditto* 225
——— Erysipelas and Injury of the Head, successfully treated by copious Bleeding. *St. George's Hospital* . . . . . 223
——— Erysipelas, treated first with Leeches, and afterwards with Stimulants. *St. Thomas's Hospital* . . . . . . 226

### ANEURISM.

Case of Diffuse Aneurism. By Mr. Guthrie. *Westminster Hospital* 25
——— Axillary Aneurism of the Right Side; Operation of tying the Subclavian Artery. *Guy's Hospital* . . . . . 135
——— Femoral and Popliteal Aneurism; external Iliac Artery tied. *Nottingham General Hospital* . . . . . . 325
——— Inguinal Aneurism, in which the external Iliac Artery was tied. *St. George's Hospital* . . . . . . . . 328

### HERNIA.

Case of Strangulated Hernia. *St. George's Hospital* . . . . 125
Strangulated Hernia in an Infant twenty days old; Operation. Treated by M. Dupuytren, at the *Hotel Dieu* . . . . . 421
Scrotal Hernia, attended with some peculiar Circumstances. *Middlesex Hospital* . . . . . . . . . . 423
Rupture of the Gut in a Hernial Sac. *Guy's Hospital* . . . . 515
Extensive Wound of the Abdomen. *Middlesex Hospital* . . . 516
Strangulated Hernia, successfully treated with Tobacco Clyster, at *St. George's Hospital* . . . . . . . . . ib.

### JAUNDICE.

Thoughts on the Pathology and Treatment of Icterus, or Jaundice. By N. Chapman, M.D. . . . . . . . . 478

### PHAGEDENIC ULCERATION.

Cases of Phagedenic Ulceration. By Mr. Earle. *St. Bartholomew's Hospital* . . . . . . . . . 34

### MALIGNANT TUMORS.

Case of Carcinoma of the Cæcum in a Boy. *St. George's Hospital* . 227
——————— Stomach. *Ditto* . . . . 230
——— Malignant Tumor within the Thorax . . . . 231

### HYDROPHOBIA.

Proposal to employ heated Air in Hydrophobia . . . . 497

### SMALL-POX AND VACCINATION.

Vaccination in Turkey. By Dr. Auban . . . . . 13
Dr. Gregory's Reply to the Animadversions of Medicus, on his Paper on Constitutional Inaptitude to Cow-Pox . . . . . 14
Cases of Vaccinia . . . . . . . . . . 30
Reply of Medicus to Dr. Gregory . . . . . . 109
Replies to Queries on Vaccination and Small-Pox. By W. Fergusson, Esq. . . . . . . . . . . . 194
Observations on Vaccination. By Medicus . . . . . 503

PAGE

GUN-SHOT WOUND.

Case of Gun-shot Wound of the Leg, in which five-sixths of the Structure at the Seat of Injury was destroyed, successfully treated, the Limb being preserved. Reported by JAMES A. WASHINGTON, M.D. House Surgeon to the *Pennsylvania Hospital* . . . . . . 508

BURNS.

Cases of Burns treated with Cotton, by Dr. ANDERSON, at the *Glasgow Royal Infirmary* . . . . . . . 511

MIDWIFERY.

On the Spontaneous Evolution of the Fœtus. By GEORGE JEWEL, Esq. 102
Cases of Uterine Hemorrhage, terminating fatally. By C. WALLER, Esq. 107
Case of Extra-Uterine Fœtation. By — TILT, Esq. . . . 378
Cases in Midwifery. By RICHARD LONG, M.D. . . . 501

MOXA.

Cases in which the Moxa was successfully applied. By Mr. R. WADE 473

MISCELLANEOUS.

Report of Cases treated at the *Aberdeen New Dispensary*, and communicated by ALEXANDER RAINY, senior Pupil . . . 26
Case of Glossitis . . . . . . . . 28
——— Sudden Death . . . . . . . . 29
——— Phthisis, with Hepatised Spleen . . . . 31
——— Melanosis . . . . . . . . 34
Observations on Black Vomit. By WM. LYONS, Esq. Staff Surgeon 100
Cases of Suffocation from the Respiration of the Fumes arising from burning Coals. *Guy's Hospital* . . . . . . 122
Observations on the Nature and Treatment of Beriberi. By WILLIAM HAMILTON, Esq. . . . . . . . . 197
Cases of Enlargement of the Liver and Spleen, treated with Iodine. *Royal Universal Infirmary for Children* . . . . 234
A Clinical Report of Chest Affection. By POWELL C. BLACKETT, Esq. 285
Case of large Tumor in the Forehead. By HENRY THOMSON, M.D. 301
Cases of Pulsating Tumors. *Middlesex Hospital* and *Glasgow Infirmary* 319
Memoir on Sprains. By M. A. PELLETIER . . . . 391
Cases of Compound Fracture, treated at the *Glasgow Infirmary* . 417
——— Nævus Maternus, cured by Vaccination. *Ditto* . . 425
Case of Tracheotomy in a Girl, nine years old. *Hospital at Berlin* . 426
Fatal Case of Intus-susception in a Child. By EDWARD HUMPAGE, Esq. 504
On the Properties of British Opium . . . . . . 505
Cases of Indolent Buboes cured by the use of the Tobacco Ointment. By Dr. GRAHAM . . . . . . . . 506
Fracture into the Knee-Joint, successfully treated. *St. George's Hospital* 517
Poisoning with Oxalic Acid, successfully treated. *St. Thomas's Hospital* ib.

### CRITICAL ANALYSES.

PAGE

An Introduction to the Comparative Anatomy of Animals. By C. G. Carus, Med. et Phil. Doct. Translated from the German, by R. T. Gore . . . . . . . . . 40

A Manual of Surgical Anatomy, by H. M. Edwards, d.m.p. Translated, with Notes, by William Coulson . . . . 54

A Treatise on the Diseases connected with Indigestion. By David Uwins, m.d. . . . . . . . . . 58

A Practical Essay on Stricture of the Rectum; illustrated by Cases. By Frederick Salmon, Esq. . . . . . 136

Observations on the Mortality and Physical Management of Children. By John Roberton, Esq. . . . . . . . 142

Diseases of the Bronchia; being the first Part of a Treatise on the Diseases of the Chest and on Mediate Auscultation. By R. T. H. Laennec, m.d. Translated from the French, by John Forbes, m.d. 145

——— Lungs; by ditto. Ditto . . . 346

Peripneumony; by ditto. Ditto . . . 442

Pathological and Practical Researches on Diseases of the Brain and the Spinal Cord. By John Abercrombie, m.d. . . 237, 330

A Grammatical Introduction to the London Pharmacopœia and Preface. By S. F. Leach, Esq. . . . . . . . 352

General View of the present State of Lunatics. By Sir A. Halliday, m.d. k.h. . . . . . . . . . 427

Practical Observations on Insanity, and the Treatment of the Insane . ib.

A Practical and Pathological Inquiry into the Sources and Effects of Derangements of the Digestive Organs. By William Cooke, Esq. 434

Disquisition on the Nature and Properties of living Animals. By G. Warren, Esq. . . . . . . . . 438

Transactions of the Association of the Fellows and Licentiates of the King and Queen's College of Physicians in Ireland. Vol. V.

On the use of the Nitrate of Silver in certain Affections of the Eye. By Isaac Ryall, Esq. . . . . . . 518

Observations on the use of Ipecacuan in Menorrhagia. By J. Osborne, m.d. . . . . . . . 520

Observations on the Peripneumonia of Children. By Thomas Cuming, m.d. . . . . . . . . 521

Observations on Cow-Pox, and on the Necessity of adopting Legislative Measures for enforcing Vaccination. By Henry Edmondston, a.m. 526

An Essay on the Diseases of the Jaws, and their Treatment. By Leonard Koecker, Surgeon Dentist . . . . . 532

---

COLLECTANEA . . *Pages* 62, 161, 248, 353, 451, 537

INTELLIGENCE . . . 84, 181, 257, 371, 471, 553

METEOROLOGICAL REGISTER 88, 184, 280, 376, 472, 560

### NAMES OF AUTHORS,

*Of whose Works, Observations, &c. either a detailed Account, or more or less general View, is given in the present Volume.*

---

PAGE

ABERCROMBIE, Dr. on the Brain and Spinal Cord (analysis of) 237, 330
ALISON, Dr. on Fever . . . . . . 68, 166
ANDERSON, Dr. his cases of Burns treated with Cotton . . 511
AUBAN, Dr. on Vaccination in Turkey . . . . . 13
BARTON, Dr. his operation for Anchylosis . . . . 77
———— his case of Anchylosis . . . . . 457
BATTLEY, Mr. his preparation of Volatile Oil of Copaiba . . 255
BIRCH, Mr. on Laceration of the Uterus . . . . 367
BLACKETT, Mr. on Chest Affection . . . . . 285
BOW, Dr. on Fever . . . . . 185, 291, 385
BROWN, Dr. on the external application of Nitrate of Silver . 458
CARPENTER, Mr. his method of preparing Narcotine . . 256
CHAPMAN, Dr. on Jaundice . . . . . . 478
COATES, Dr. on Delirium Tremens . . . . . 356
COOKE, Dr. on the Digestive Organs (analysis of) . . . 434
COOPER, Mr. BRANSBY, his case of Ligature of the Subclavian Artery 135
COULSON, Mr. his translation of EDWARDS' Anatomy . . 54
CULLEN, Dr. on the causes of fatal terminations in Bronchotomy . 249
CUMING, Dr. on the Peripneumonia of Children (analysis of) . 521
DUPUYTREN, M. his method of treating Stricture of the Lachrymal Duct 254
EARLE, Mr. his case of Inflamed Vein . . . . 22
———— his cases of Phagedenic Ulceration . . . 34
———— on Paraplegia . . . . . . . 65
EDMONSTON, Mr. on Cow-Pox (analysis of) . . . . 526
FERGUSSON, Mr. on Vaccination in Africa . . . . 194
GIBBES, Sir G. on Life . . . . . . . 9
GORE, Dr. his translation of CARUS'S Comparative Anatomy . 40
GRAHAM, Dr. on the application of Tobacco Ointment to Indolent Buboes . . . . . . . . 506
GREGORY, Dr. his reply to MEDICUS . . . . 14
GUTHRIE, Mr. his case of Diffuse Aneurism . . . . 25
HALFORD, Sir H. on Tic Douloureux . . . . . 452
HALLIDAY, Sir A. analysis of his General View of the present State of Lunatic Asylums . . . . . . 427
HAMILTON, Mr. on Beriberi . . . . . . 197
HENRY, M. his method of preparing Sulphate of Quinine . . 255
HORNER, Dr. on the Mucous Membrane of the Stomach and Intestines 113, 208, 304
HUMPAGE, Mr. his case of Intus-susception . . . . 504
JAMES, Dr. on the Circulation . . . . . 1
JENNINGS, Mr. on the Iris . . . . . . 281
JEWEL, Mr. on spontaneous Evolution of the Fœtus . . 102
KOECKER, Mr. on Diseases of the Jaw (analysis of) . . . 532
LAENNEC, M. on Diseases of the Bronchia (analysis of) . . 145
———— on Peripneumony (analysis of) . . . 442
———— on Diseases of the Lungs (analysis of) . . 346
LAWRENCE, Mr. on Dislocations of the Vertebræ . . .
LEACH, Mr. F. his introduction to the London Pharmacopœia . 352
LISTON, Mr. case in which he restored the Canal of the Larynx, when nearly obliterated . . . . . . . 248

PAGE
LONG, Dr his cases in Midwifery . . . . . 501
LYONS, Mr. on Black Vomit . . . . . . 100
MAC DOWELL, Dr. on the Bones . . . . . 403, 490
MACKINTOSH, Sir JAMES, his Speech on the Difficulty of procuring Subjects for Dissection . . . . . . 554
MACLEOD *v.* WAKLEY, trial for Libel . . . . 257
MERRIMAN, Dr. on the Duration of Pregnancy . . . 81
MIGUEL, M. his case of open Foramen Ovale . . . . 353
MOTT, Dr. of New York, his case of successful Ligature of the common Iliac Artery . . . . . . . 463
ORTON, Mr. his case of Amputation at the Hip-Joint . . 80
OSBORNE, Dr. on Ipecacuanha in Menorrhagia (analysis of) . 520
PARISET, Mr. his remarks on Dentition . . . . 353
PELLETIER, Mr. on Sprains . . . . . . 391
ROBERTSON, Mr. on the Mortality, &c. of Children (analysis of) . 142
ROLFE *versus* STANLEY, remarks on the verdict in the case of . 357
RYALL, Mr. J. on the use of Nitrate of Silver in certain Affections of the Eye (analysis of) . . . . . . 518
SALMON, Mr. on Stricture of the Rectum (analysis of) . . 136
SMITH, Professor, on Necrosis . . . . . 461
STRATFORD, Mr. on Cataract . . . . . . 89
TENTERDEN, Lord, his charge to the Jury in the cause MACLEOD *v.* WAKLEY . . . . . . . . 276
THOMAS, Dr. C. his case of Paraplegia . . . . 37
THOMSON, Dr. H. his case of Tumor on the Forehead . . 301
TILT, Mr. his case of Extra-uterine Fœtation . . . 378
TRAVERS, Mr. his cases of Fracture of the Skull . . . 129
UWINS, Dr. analysis of his Treatise on Indigestion . . . 58
VOGEL, Dr. his observations on the Sense of Smell . . . 62
WADE, Mr. his cases of Epilepsy and Paralysis successfully treated with Moxa . . . . . . . . 473
WALLER, Mr. his case of fatal Uterine Hemorrhage . . . 107
WARDROP, Mr. observations on his method of Tying the Vessel in Aneurism . . . . . . . . 172
WARREN, Mr. analysis of his Disquisition on the Nature and Properties of Animals . . . . . . . . 438
WASHINGTON, Dr. his case of Gun-shot Wound of the Leg . . 508
WELBANK, Mr. on the distinction between Syphilis and Phagedena 77

J. and C. Adlard, Printers, Bartholomew Close.

